# Supplementary material for: Neural ensemble dynamics in dorsal motor cortex during speech in people with paralysis
Source: eLife. 2019 Dec 10;8:e46015. doi: 10.7554/eLife.46015 (PMC6954053; doi:10.7554/eLife.46015)
Supplement: Transparent reporting form [file elife-46015-transrepform.docx]

***eLife’s* transparent reporting form**

We encourage authors to provide detailed information *within their submission* to facilitate the interpretation and replication of experiments. Authors can upload supporting documentation to indicate the use of appropriate reporting guidelines for health-related research (see [EQUATOR Network](http://www.equator-network.org/%20)), life science research (see the [BioSharing Information Resource](https://biosharing.org/)), or the [ARRIVE guidelines](http://www.plosbiology.org/article/info:doi/10.1371/journal.pbio.1000412) for reporting work involving animal research. Where applicable, authors should refer to any relevant reporting standards documents in this form.

If you have any questions, please consult our Journal Policies and/or contact us: [editorial@elifesciences.org](mailto:editorial@elifesciences.org).

**Sample-size estimation**

- You should state whether an appropriate sample size was computed when the study was being designed
- You should state the statistical method of sample size computation and any required assumptions
- If no explicit power analysis was used, you should describe how you decided what sample (replicate) size (number) to use

Please outline where this information can be found within the submission (e.g., sections or figure legends), or explain why this information doesn’t apply to your submission:

The number of trials for each speaking task dataset, orofacial movements task dataset, and movement comparisons task dataset is provided in the relevant task description in the Methods*.*

The number of trials for the breathing analysis is provided in the Figure 2–figure supplement 2 legend.

**Replicates**

- You should report how often each experiment was performed
- You should include a definition of biological versus technical replication
- The data obtained should be provided and sufficient information should be provided to indicate the number of independent biological and/or technical replicates
- If you encountered any outliers, you should describe how these were handled
- Criteria for exclusion/inclusion of data should be clearly stated
- High-throughput sequence data should be uploaded before submission, with a private link for reviewers provided (these are available from both GEO and ArrayExpress)

Please outline where this information can be found within the submission (e.g., sections or figure legends), or explain why this information doesn’t apply to your submission:

The key results were replicated in two human participants, which is the *de facto* standard in the field for human and NHP neurophysiology. This is described in the Results and Methods sections.

**Statistical reporting**

- Statistical analysis methods should be described and justified
- Raw data should be presented in figures whenever informative to do so (typically when N per group is less than 10)
- For each experiment, you should identify the statistical tests used, exact values of N, definitions of center, methods of multiple test correction, and dispersion and precision measures (e.g., mean, median, SD, SEM, confidence intervals; and, for the major substantive results, a measure of effect size (e.g., Pearson's r, Cohen's d)
- Report exact p-values wherever possible alongside the summary statistics and 95% confidence intervals. These should be reported for all key questions and not only when the p-value is less than 0.05.

Please outline where this information can be found within the submission (e.g., sections or figure legends), or explain why this information doesn’t apply to your submission:

Trial counts (N) for every dataset are provided in the Methods section.

When measurements’ mean and SD (e.g., spike waveform shape) or SEM (e.g., firing rates) are shown, they are described in figure legends.

The statistical test used for each comparison (e.g., shuffle tests, rank-sum tests) are described in the relevant Methods statement.

Raw data are generally not shown since the number of samples were >> 10. Example raw data are shown in several figures.

(For large datasets, or papers with a very large number of statistical tests, you may upload a single table file with tests, Ns, etc., with reference to sections in the manuscript.)

**Group allocation**

- Indicate how samples were allocated into experimental groups (in the case of clinical studies, please specify allocation to treatment method); if randomization was used, please also state if restricted randomization was applied
- Indicate if masking was used during group allocation, data collection and/or data analysis

Please outline where this information can be found within the submission (e.g., sections or figure legends), or explain why this information doesn’t apply to your submission:

This study was conducted with two BrainGate2 participants in the context of an FDA IDE pilot clinical trial of an intracortical bra-computer interface. No treatment was provided or evaluated in the course of this particular speech study; as such, there was no allocation into groups. Only two participants were actively enrolled in the BrainGate2 trial during this research, and both were included in this particular study; no participant selection/rejection was performed.

**Additional data files (“source data”)**

- We encourage you to upload relevant additional data files, such as numerical data that are represented as a graph in a figure, or as a summary table
- Where provided, these should be in the most useful format, and they can be uploaded as “Source data” files linked to a main figure or table
- Include model definition files including the full list of parameters used
- Include code used for data analysis (e.g., R, MatLab)
- Avoid stating that data files are “available upon request”

Please indicate the figures or tables for which source data files have been provided:

Raw neural and audio data has not been provided out of respect for the clinical trial participants’ expectation of privacy. Code and processed source data underlying each figure are provided.
